# Supplementary figures and images for: Generation of porcine PK-15 cells lacking the Ifnar1 or Stat2 gene to optimize the efficiency of viral isolation
Source: PLoS One. 2023 Nov 8;18(11):e0289863. doi: 10.1371/journal.pone.0289863 (PMC10631621; doi:10.1371/journal.pone.0289863)

# S1 Raw image. Raw images of western blotting

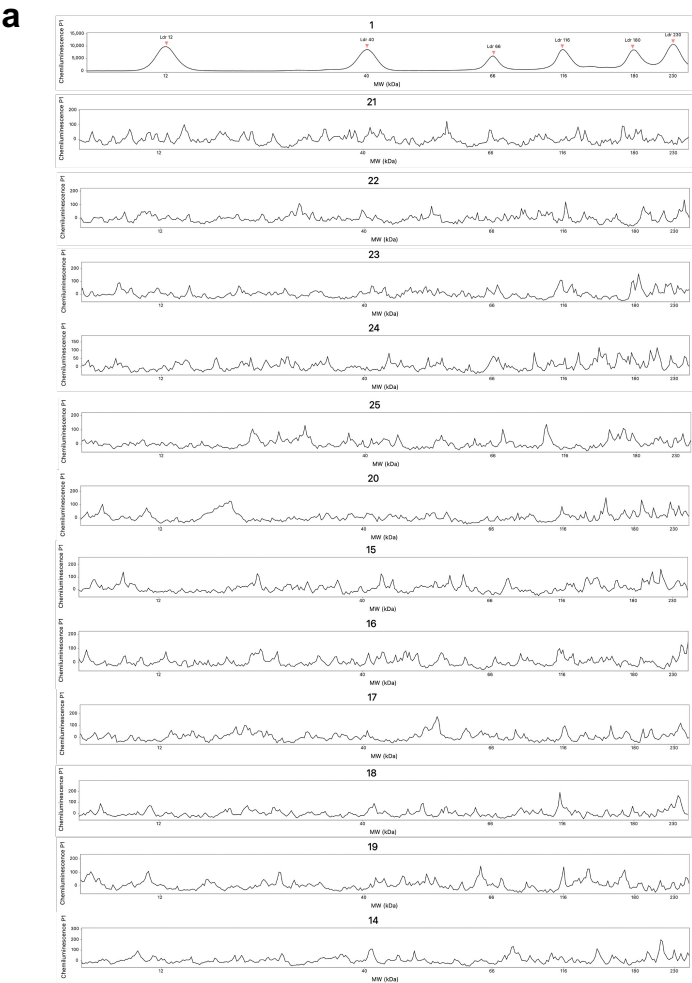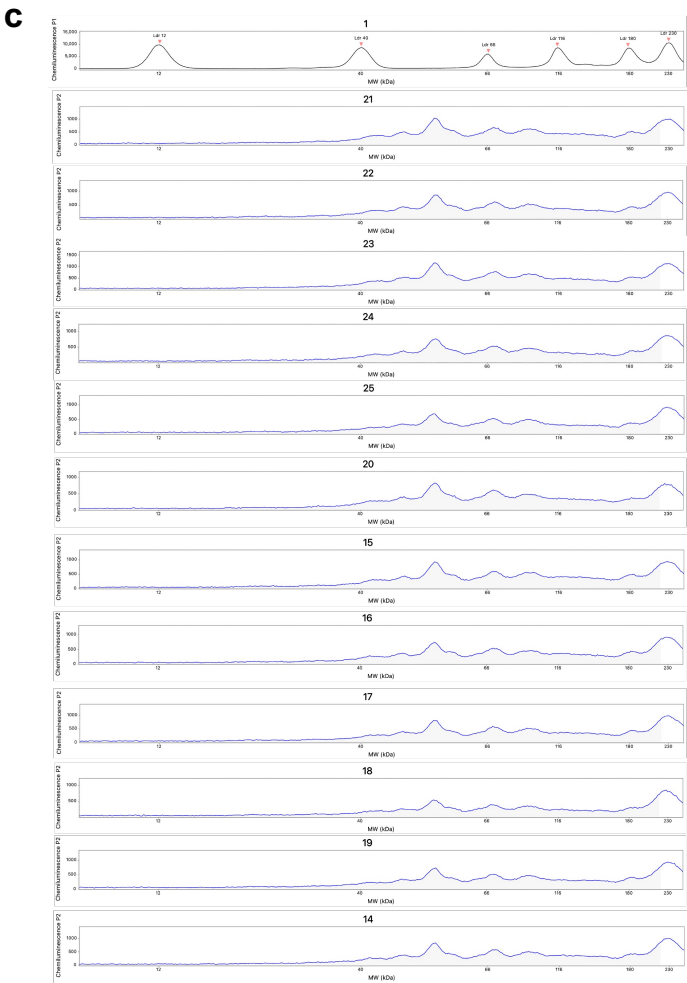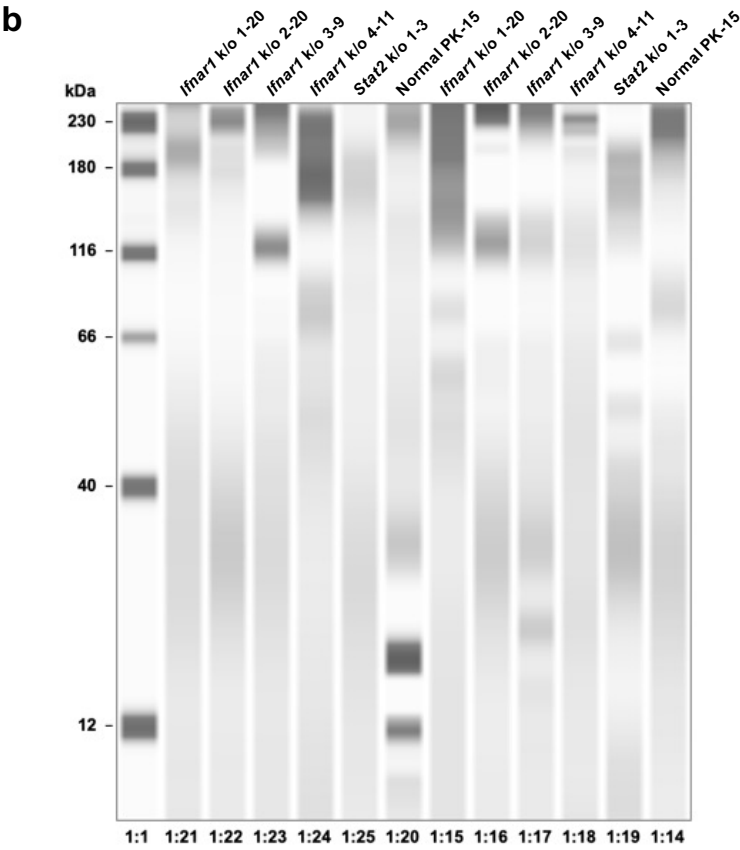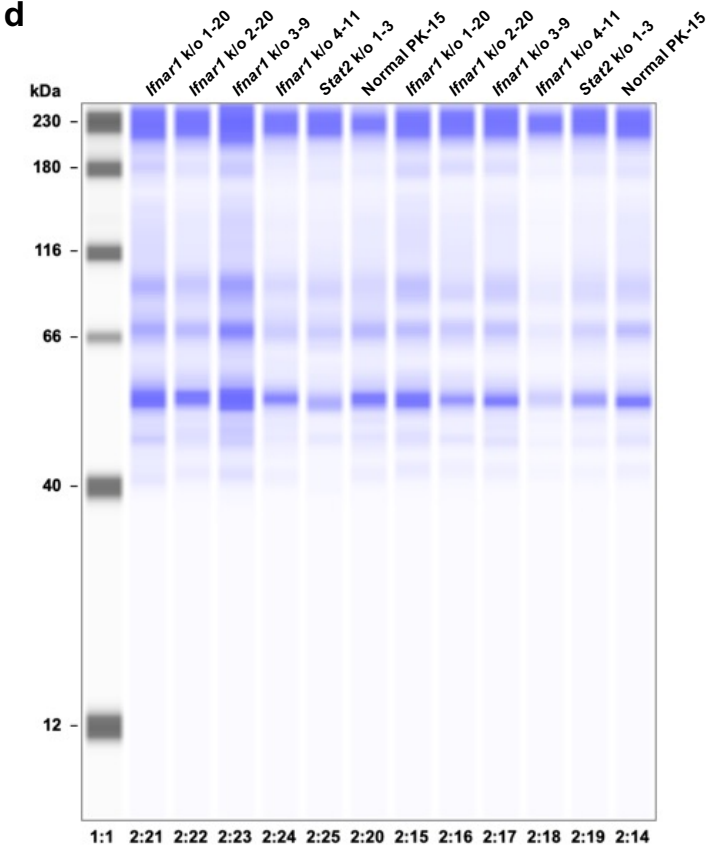

Supplement: S1 Raw image — (PDF) [file pone.0289863.s002.pdf]
